# A Treatise on the Diseases of the Bones

**Published:** 1850-01

**Authors:** 


### REVIEWS.

ART. V.—*A Treatise on the Diseases of the Bones.* By EDWARD STANLEY, F. R. S., President of the Royal College of Surgeons of England, and Surgeon to St. Bartholomew's Hospital. Philadelphia: Lea & Blanchard. 1849.

In our former notice of this valuable book, we reached the section on suppuration. On this interesting subject, Mr. Stanley is very lucid and practical, and his remarks and facts deserve the attention of the general practitioner. We shall endeavor to present our readers with the principal matter of his remarks on this subject.

Suppuration in the bone is conveniently distinguished into the circumscribed and diffused abscess. The circumscribed abscess is usually situated near to, or within the articular ends of long bones; but it is sometimes formed in the middle of their shafts. "Generally, at the seat of the disease, the coverings of the bone are tender, a constant aching is felt in the bone, with paroxysms of acute pain and severe constitutional derangement. In some instances there is a remission of the symptoms so complete, and for such a length of time that the patient seems to be well: but the pain in the bone, from the confinement of matter within it, has returned." In one instance Mr. Stanley traced the remission of the symptoms to the formation of a passage in the wall of the abscess, permitting the escape of the matter from the interior of the bone into the parts around it. In some instances a circumscribed abscess enlarges much beyond the natural limits of the bone. This is in consequence of the combined actions of absorption on the inside of the abscess, and of osseous deposit on the outside, and the osseous walls may acquire any degree of thickness according to the predominance of absorption in one direction, or of osseous deposit in the other. In the museum of the Royal College of Surgeons in Edinburgh, there is an instance of a large osseous cyst originating in the head of the tibia the walls of which are more than an inch thick. A circular aperture, about an inch in diameter, extends through the wall of the cyst, into which the patient used to introduce a wooden plug, and withdraw it to allow the escape of matter, on feeling pain from distension of the cyst. In this way, from sixteen to twenty-four ounces of purulent fluid were every day discharged from the cyst." "Another osseous cyst, in the same museum, within the lower part of the femur, is twenty inches in circumference."

"A diffuse suppuration through the cancellous and medullary tissue of bone is usually a most formidable disease, leading to destruction of the bone and of the soft parts around it, with the most severe constitutional derangement."

The usual causes of suppuration in bone are local injuries, long exposure to cold and moisture, fever, sloughing of the soft parts, after amputation, and compound fractures. The general practitioner should remember the fact that suppuration may take place in the medullary tube of a bone involved in compound fracture, and should also remember that such an evil is scarcely possible, if the proper application is attended to. A contemplation of the disasters that attend suppuration in the bone should lead the judicious practitioner to take care that the evil shall not come from mal-practice.

Suppuration in bone may end in necrosis, and the evidence that the necrosis is from that cause, is "the deposit of purulent fluid or lymph within the bone, which is not a character of necrosis arising under any other circumstances."

The diagnosis of suppuration in bone is somewhat difficult. The distinctions between the neuralgic affection of bone, inflammation of its tissue, and suppuration, are not so perfect as to secure the physician against error. The necessity for correct diagnosis is obvious from the fact that perforation of the bone must be resorted to in order to remove the purulent collection. The symptoms accompanying the circumscribed abscess in bone are not in general such as bespeak the nervous or hysteric character of the disease, but, Mr. Stanley says, we must not too confidently rest upon this.

Inflammatory affections of bones most frequently arise in the shafts of the bones, while the circumscribed abscess rarely occurs elsewhere than in their articular ends. But Mr. Stanley points out a difficulty here that should not be forgotten. He says: "Part of the shaft of a long bone has gradually enlarged from inflammation in its tissue; then upon the occurrence of a fresh paroxysm of inflammation, increased swelling of the bone has ensued with such severity of pain as to have led to the supposition that there must be matter confined within it." These symptoms may arise from tension and stretching of the inflamed periosteum over the swollen bone.

In the commencement of the remarks on the treatment of suppuration in bone, Mr. Stanley very properly pays a well merited tribute to Sir Benjamin Brodie for "ascertaining the circumstances which indicate the confinement of a small quantity of matter in a circumscribed cavity within bone." By applying this knowledge in perforating the bone, and "thus obtaining an outlet for the matter with immediate and complete relief," Sir Benjamin has saved limbs, which, without this improvement in surgery, would have been consigned to the amputating knife.

In applying the trephine for the evacuation of pus from bone, the rule is to select the chief seat of pain as the place for the use of the trephine. After this instrument has penetrated as far as the judgment of the surgeon may decide prudent and necessary, if no matter should appear, the cancellous texture should be pierced with a small perforator, around the opening made by the terphine. It must be remembered that the smallest quantity of purulent fluid confined within a bone has been the source of very severe suffering; and, when mixed with blood, which generally escapes freely from the inflamed cancellous texture around the abscess, the purulent fluid might not be distinctly recognized. The character of the fluid escaping from the bone should, therefore, be closely scrutinized. The application of these principles to collections of matter in the bones of the cranium requires great care and judgment. Mr. Abernethey's high authority may easily lead the practitioner into difficulty, for symptoms that may be supposed to denote the existence of pus in the diploe may arise from inflammation of the membranes of the brain. Even in cases of purulent accumulation in the diploe, seen by Mr. Stanley, where the trephine was used, it was of no avail; the accompanying inflammation in the membranes of the brain proved fatal. Suppuration in these bones, and necrosis require careful management. An expectant treatment is the proper one.

From the masterly exposition of the subject of suppuration in bone, Mr. Stanley passes to caries, in which term is expressed those changes that are consequent on chronic suppuration in the cancellous texture of bone.

Caries is distinguished into the simple, scrofulous, syphilitic, and phagedenic varieties. It ensues from comminuted fractures of the cancellous texture, or from such injuries as the crushing of this texture by a bullet; it also ensues from the influence of the scrofulous diathesis, and from the action of the syphilitic poison; and there are cases of it not referable to any distinct cause.

We quote Mr. Stanley's description of the phenomena of caries. He says, they are: "inflammation extending from the bone to its investing soft parts, these become swollen, thickened, and tender; abscesses are formed in them, which contract into fistulous passages leading to the diseased bone. The periosteum covering the diseased bone becomes thickened, very vascular, and readily separable from it. The bone itself is at first very vascular, then its cells become filled with a reddish brown fluid, apparently a mixture of blood and pus, and occasionally combined with oily particles. Absorption of the bone, but chiefly of its animal part, ensues; that which remains is porous and fragile, and of a grey, brown, or black color, probably from decomposition of the matter within its cells; to which cause, likewise, the fetid odor of the matter discharged through the fistulous passages may be ascribed. The diseased bone may gradually disappear, either by ulceration, or by its discharge in fragments through the fistulous passages in the surrounding soft parts. Ulceration, in some instances, commences within the bone, hollowing it out, and reducing it to a thin shell; in others, the ulcerative process commences in the outer surface of the bone, and extends progressively inwards. Whilst these changes are in progress, granulations, very loose and spongy, and bleeding on the slightest touch, often arise from the diseased bone, filling the cavities in its interior, and protruding through the fistulous passages in the soft parts covering it."

But in bones that are deeply situated, caries is often accompanied by very little evidence of inflammation, either in the bone or investing soft parts. Thus the scrofulous caries of the spine often advances to the destruction of the bodies of many vertebra, without tenderness in the bones or in the soft parts investing them.

The difference between caries and circumscribed abscess is tolerably well marked. Circumscribed abscess is rarely followed by the formation of a fistulous channel in the walls of the bone and adjacent soft parts, affording outlet to the matter. In caries and internal necrosis, such a channel is almost invariably formed in the walls of the bone and their investing soft parts; through which channel, in caries, matter is discharged from the diseased bone; and in necrosis, from the inflamed cancellous texture around the dead bone.

The diagnosis between caries and internal caries is not well marked, and practically it is not important that it should be. The object of the treatment is the same in both cases—the removal of the diseased, or dead bone. Caries and necrosis often exist together in adjacent portions of bone, as in the instances where suppuration ensues in the cancellous texture adjacent to dead bone. The portion of bone which has been long carious often perishes, and thus the caries becomes changed into necrosis. These circumstances lessen the value, says Mr. Stanley, of a diagnosis of these affections by their external character.

Inflammation precedes suppuration and the consequent disorganization of the cancellous texture of bone which constitute caries. The inflammation is of a languid character, and though depletory remedies will not arrest the inflammation, they are serviceable by diminishing the inflammation of the soft parts around the diseased bone.

Nature does much for the relief of caries, and but few cases occur in surgical practice that require the exercise of a finer judgment than caries of the various bones. For instance, when the astragalus is in a carious condition, it is difficult to determine whether the disease is confined to it, or has extended to the adjacent bones and soft parts. Similar difficulties present themselves in caries of the cuniform bones. Internal necrosis and circumscribed abscess in the tarsus present similar considerations. Such cases present the same thickening of the surrounding soft parts, and fistulous passages in them are observed as in caries. The difficulty is to determine how far the diseased action extends. Most of the operations for the removal of carious bone occur in the hand or foot; and in the performance of them the endeavor should be to remove no more bone than may be unsound.

Incisions through the soft parts over carious bone are of use in affording free outlet to the matter, and thus checking its diffusion through the bone. Local applications to carious bone must be of a soothing character. Stimulent applications will do no good, and may produce fresh attacks of inflammation in the bone and thus extend the caries.

There are cases of caries not suited to any operative proceedings. Peculiarities of constitution, and old age are the points to be considered in such cases. Depletory and sedative remedies, constitutional and local are applicable here, and iodide of potassium is useful in arresting the inflammation of the periosteum, covering the diseased bone. Quietude is of great value in all cases of caries, and its necessity cannot be too forcibly impressed upon the mind of the patient, nor too rigorously enforced by the surgeon.

*Ulceration of Bone.*—Under this head we find little that is beyond what has been recorded by Hunter, Weidman, and others, and we shall not dwell upon the characteristics of the disease. The treatment recommended by Mr. Stanley is practical and as useful perhaps as any that has been presented to the profession. The local remedies should be of a soothing nature, but sometimes astringents and mild stimulants are serviceable for the promotion of the growth of healthy granulations from the bone. Strongly stimulating applications to ulcerated bone are objectionable, as they are likely to excite fresh attacks of inflammation in the bone, and may thus change the character of the disease, by giving rise to caries or necrosis in the ulcerated bone. Mr. Stanley says: "It is not to be doubted that a powerful counter-irritant, as the moxa or caustic issue, applied to the integuments over a joint, the bones of which are ulcerated, can arrest the ulcerative process." "It is certain, that in ulcerative disease of the bones of the spine, as also in joints, a powerful counter-irritant will rid the part of the severe pain which characterizes the ulceration of bone in its active form."

It may not be amiss to call attention to a state of things, in which symptoms of inflammation of the bone are so well simulated that an unpracticed mind might be led into a serious error. A man in apparent health is suddenly seized with an excruciating pain in the hip-joint. Every attempt at sneezing or coughing produces such an amount of suffering in the joint, that the patient sedulously guards himself against everything of the kind. We have seen this form of disease occasionally in Louisville, and have often witnessed the severe trials of the sufferer in his attempts to prevent sneezing or coughing. We are indebted to Mr. Abernethy for a description of this disease. There is really no affection of any of the parts of the joint—the derangement is in the stomach, and that organ, instead of complaining itself, makes the hip-joint express the suffering. Mr. Abernethey's invaluable treatise on the "Constitutional Origin of Certain Local Diseases," reveals many singularities of this kind, and shows the proper treatment for such a case as we have described. A simple emetic is usually sufficient for the relief of such a case, but sometimes, an alterative course of blue pill is necessary to accomplish permanent relief. The emetic, however, removes the pain in the hip instantaneously, and we have seen all traces of suffering disappear as soon as nausea was produced.

In our regular analysis of Mr. Stanley's admirable work, we have reached the subject of necrosis, a theme better calculated than almost any other to excite the admiration of the surgeon, and to demand the use of his contemplative powers. The chemist, ardent in the love of his science, proud of its great achievements, its wonderful progression, and bewildered by what it has accomplished, sometimes permits himself to fancy that the object of his devotion may yet succeed in making all the materials useful to human life, from the elementary substances of which they are composed. The physiologist in contemplating the reproductive powers of the vital principle in animal life; in watching the curious methods by which nature regenerates parts that have been destroyed, may be almost excused if he indulges the fancy, that, in the future progress of science, vital processes may be better understood than at present, and that an art may be discovered by which the various organs and tissues of the system, as they wear out by action, may be rejuvinated and presented to the vital power for a new race in existence. The vital principle never grows old; it tenaciously adheres to its connection with organism as long as the material is susceptible of its impressions.

In surveying a subject of such importance as necrosis much useful matter relative to practical points in other subsystems, beside the osseous one, must spring up in the mind of the attentive student. While we narrowly survey nature in her connection with necrosis, inspiration may be caught from her processes for a widely extended use of the lessons thus taught. The whole realm of nature belongs to the physician, and he alone is accomplished in his science, who has made an intimate acquaintance with that extensive domain. All the sciences are the servitors of medicine, and he is most wealthy in the treasures of healing knowledge, who has the largest number of these servitors usefully employed. But we must proceed with the subject of necrosis.

Mr. Stanley says: "Necrosis of the shaft of a long bone in the ordinary mode of its occurrence, is an example of the simple death of bone without change in its structure; but when necrosis has been preceded by chronic inflammation, the dead bone will be hardened; or after other diseased changes, it may be soft and fragile."

"Dead bone usually presents a yellowish white color, consequent on the blood being withdrawn from its vessels, but it often becomes of a brown or black color. The color of a dead bone appears to be influenced by its exposure to the atmosphere. It is often brown or black to the extent only that it is uncovered; the change of color does not extend below the surface of the bone, except in the instances of its texture being porous."

*The portion of bone usually affected with Necrosis.*—Necrosis occurs less frequently in the cancellous than in the compact tissue of bone. Owing to this resisting power in the cancellous texture, rising from greater vital endowment, necrosis in the shaft of a bone, *rarely* extends to its articular ends.

*Bones most generally affected.*—The tibia is more liable to necrosis than any other bone, and the liability of the other bones is in the following order; the femur, the humerous, flat cranial bones, lower jaw, last phalanx of the finger, clavicle, ulna, radius fibula, scapula, upper jaw, pelvic bones, and the ribs. An attention to this order of liability to necrosis may give some aid to diagnosis. A certain class of symptoms occurring in the tibia would lead the surgeon to suspect necrosis more readily than if those symptoms presented themselves in connection with the ribs. The tibia appears to be the most general point for invasions of disease. Necrosis is of frequent occurrence in the cancellous texture of the head of the tibia, so frequently, indeed, that there seems to be something in the structure and functions of the head of that bone which especially disposes it to the invasion of disease, for here not only necrosis, but also abscess makes its attack, and here, also, the malignant affections of bone are frequently developed. This fact, to which the records of medicine furnish a cloud of witnesses, should teach the surgeon a practical lesson in selecting his point for an amputation of the leg. There is a strong disposition on the part of the surgeon to save the knee joint to aid the use of an artificial leg, but we cannot help thinking this is misplaced kindness. All scientific manufacturers of artificial legs know that they can make a limb of this kind much more comfortable and useful when the amputation is made above the knee than when it is below it. When the tibia and fibula lose that *point d'appui*, they had in the ancle joint, their presence in the stump is a serious evil; they grind and annoy the soft parts, and give scarcely any support to the knee joint; none indeed that compensates for their presence. The weakest point in the system for the invasion of disease of the bones, in this state of things, presents additional facilities for disease. In view of all these facts, we think that amputations of the leg, should, as almost a universal rule be made above the knee.

*Causes of Necrosis.*—"The recognised causes of necrosis are cold, injury by violence, rheumatism, scrofula, syphilis, and fever. Some of these causes act directly on the periosteum; others on the medullary tissue; and others on the bone itself. All these tissues may be affected by inflammation in a case. Paronychia, followed by necrosis of the last bone of the finger, probably commences in inflammation of the periosteum." Of this fact, stated by Mr. Stanley, we think there can be no doubt. We do not remember any case that has come under our observation, in which the peculiar odor belonging to suppuration of bone was manifested in the first discharge from a paronychia, and in which the pus was not devoid of the usual appearance of that arising from inflammation of the osseous tissue.

In addition to the causes of necrosis mentioned above, Mr. Stanley calls attention to certain occasional agencies that come into play. Salivation is probably the most frequent cause of necrosis in the lower jaw. Another of these occasional causes of necrosis, both in the upper and lower jaws is connected with the manufacture of lucifer matches. Phosphorous acid vapor is the poisonous agent in these cases. On this subject a volume of testimony has already accumulated, and the disastrous effects have been seen so often, that this necrosis assumes an important position. Why the phosphorous acid vapor acts especially on the jaw bones is a matter for investigation. At present we can give no more reason for it, than for the fact that in arsenical poisoning, from a local application to the extremities of the body, ulcerations are found in the stomach just as they are when the poison is taken directly into that organ.

While speaking of the agency of the phosphorous acid vapors in producing necrosis of the jaws, Mr. Stanley states some facts that are of interest upon a Western subject. We allude to the cause of milk-sickness. With a laudable solicitude for the advancement of medical science, in the mitigation or removal of human suffering, some well informed medical men have investigated the effects of arsenic upon dairy animals, under the impression that milk-sickness was caused by arsenical vapor. But the facts detailed by Mr. Stanley show that this is an error. At the copper works at Swansea, where copper is melted, arsenical fumes are evolved to such an extent as to destroy all animal and vegetable life within their influence. Cows, in that region, become disabled from disease in the bones, a fact which has never been noticed as an attendant upon the disease in those animals which were distinctly recognized as the source of milk-sickness in the human subject.

But that phosphorous acid vapor should display its poisonous energies in producing necrosis of the maxillary bones, and that arsenical fumes should direct their powers on the general osseous system, enlarging the bones, and covering them with deposits of unhealthy osseous matter, are curious facts that need elucidation.

But we must examine now into the action of the causes of necrosis we have mentioned, especially in its attacks upon some of the small bones. The tuber ischii, or the trochanter major are destroyed with their soft coverings, in feeble systems, from inâbility to sustain the pressure to which the parts are subjected.

In scrofulous children, necrosis occurs in portions of bones having a cancellous texture ; but in these instances the death of the bone is usually preceded by suppuration through its cells.

Syphilis produces its effects mostly on compact osseous texture, and in portions of bones which have their soft coverings, as the flat cranial bones, the front surface of the tibia, and the posterior border of the ulna, near the olecranon. Syphilitic poison produces the immediate and complete death of the surface of the bone it attacks.

Rheumatic inflammation of the periosteum is another cause of necrosis in small portions of bone. This most frequently occurs in the periosteum of the trochanter major, and adjacent parts of the neck and shaft of the femur. Here suppuration may assume a formidable state of things, by the matter bursting into the hip-joint.

*Fistulous passages.*—In many instances of necrosis attacking small portions of bone, it is not possible to make out satisfactorily the history of the disease, in respect to the cause of the death of the bone. The common feature, however, of all these cases should be impressed upon the mind—the existence of one or more fistulous passages in the soft parts, often circuitous in their course, the orifices of which in the integuments are often at a great distance from the piece of dead bone to which they lead.

A fistulous passage in the soft parts adjacent to dead bone, whether this be superficial or deep, is an almost unvarying occurrence. In superficial necrosis, abscess forms in the adjacent soft parts, and the matter tracks its way to the integuments, through which it is discharged, the abscess then contracting to the fistulous passage which leads to the dead bone. In deep necrosis, implicating only the inner lamella of a bone, a fistulous passage is formed, first in the adjacent walls of the bone, and then in the soft parts covering it. This is the usual course of things, but enough departures from it have been noticed to show that we must be guarded on this point.

*Symptoms of Necrosis.*—When necrosis occurs in the outer lamella of a bone, it is not easily distinguishable from other inflammatory affections of periosteum or bone. The phenomena ordinarily attendant on its progress are: separation of the periosteum from the dead bone; inflammation of the periosteum, followed by suppuration beneath it, and by ulceration of it with the investing soft parts, affording outlet to the matter, and terminating in a fistulous passage leading to the dead bone.

Necrosis in the inner lamella has its characteristic signs. The first symptom, generally, is pain deep in the bone, but sometimes, fever, continuing many days or even weeks, and then subsiding, with the commencement of pain in the bone, is the first symptom. The death of the inner lamella is followed by suppuration in the adjacent cancellous texture, also by the formation of a narrow fistulous passage in its walls. While these changes are going on within the bone, inflammation arises in the soft parts covering it, accompanied by a soft circumscribed swelling of the integument, from which, when punctured or allowed to burst, matter will be discharged. Through the abscess in the soft parts, the orifice of the fistulous passages may be detected, but it may be so small as not to be found, which may lead to difficulty in determining the real nature of the disease. Mr. Hey was led to suspect an aperture in the walls of the bone from the amount of matter discharged, being greater than the surface of the outer wound should have furnished, and Mr. Stanley says his observations have enabled him to confirm the value of the suggestion.

Necrosis of the shaft of a bone in its whole thickness, whether in a part of its length, or occupying the whole of it, presents the following features: in some instances febrile disorder precedes the death of the bone, but generally a sudden attack of pain deep in the bone is the first symptom; this is quickly followed by excessive inflammation in the soft parts, accompanied by fever. As soon as the necrosis occurs the periosteum loses its hold on the bone, and when separated from the bone, the periosteum becomes acutely inflamed. The inflammation extends through the other tissues to the integuments where it presents a phlegmonous or erysipelatous character. The terminations of this inflammation may be serous effusion, attended with mere enlargement, or in the deposit of fibrin consolidating the textures, or in suppuration beneath the periosteum, and in the intermuscular cellular tissue giving rise to one large abscess, or to many small ones in different parts of the limb. Incisions are usually required for the discharge of the matter from these abscesses; and then, unless there is an unusual source of irritation maintaining the suppuration, the quantity of matter secreted will gradually diminish, and some of these passages will be closed, whilst others remaining fistulous will lead to the cavity in which the dead bone is enclosed.

Necrosis in the shaft of a bone, through the circumference and thickness of its walls, is usually accompanied with the death of its medullary tissue to a corresponding extent. But there are instances of necrosis arising only on the surface of the bone, in which the walls of the bone perish, while the medullary tissue preserves its vitality.

Necrosis of the shaft of a bone near its articular end is often followed by the extension of inflammation to the adjacent joint: thus, disease in the knee-joint has ensued from a necrosis either in the femur or tibia near its articular end. And destruction of a joint may occur from necrosis, by the abscess in the soft parts around the dead bone, bursting into the joint. Attention should be directed to these cases. Amputations have been performed for diseases of the joints, and they might have been avoided if it had been known that a small piece of loose bone, imprisoned within the living bone, was near the joint, and could be removed, by which the limb might have been saved.

When the necrosis is of small size, or only a small part of the bone has perished in a patient who is not of an irritable habit, the inflammation in the soft parts is apt to be of a mild character.

Necrosis of a part of the cancellous texture within the articular end of a bone is a formidable disease, as it often destroys the adjacent joint. This necrosis most frequently occurs in the head of the tibia, in persons between the ages of fifteen and twenty years, often without appreciable cause, but most frequently from a blow upon the knee. The great danger here, arises from the inflammation being in the neighborhood of the joint. This point is so important, that instead of giving a mere synopsis of Mr. Stanley's observations, as we have been doing, we shall in this case give way to a quotation from him. He says: "The dead portion of the cancellous texture within the head of the tibia is rarely as large as a hazle nut; its situation varies, being nearer to the front or back wall of the bone, or to its upper articular surface, in one case than in another. The separation of the dead from the living bone usually takes place in an early stage of the disease. The piece of dead bone is then loose in a suppurating cavity, lined by a thick and very vascular membrane, from which pus is secreted more or less abundantly, according to the degree of excitement in the surrounding parts. The dead bone, being an irritant, excites constant pain, with successive attacks of inflammation, in the adjacent living bone; also in its periosteum and in the knee-joint. Consequently, the surrounding cancellous texture becomes hardened, and narrow ulcerated passages, which become fistulous, extend in various directions through the hardened bone to the front or back wall of the tibia, or towards its upper articular surface. If these ulcerated passages extend through the wall of the bone to its periosteum, matter will then accumulate beneath the periosteum, or ulceration of the periosteum will ensue, permitting the extension of the abscess into the surrounding soft parts. If one of the ulcerated passages in the bone should extend upwards to its articular surface, and then open into the knee joint, rapidly destructive inflammation of it will ensue. During the progress of the disease, the periosteum investing the head of the tibia and the cellular tissue around it, become thickened and indurated, thus giving to the bone the appearance of actual enlargement."

We resume the consideration of the progressive steps of necrosis.

The term exfoliation applies to that process in bone, which we call sloughing in the soft parts. In both cases, a soft vascular tissue marks the line of separation between the living and dead parts. The granulations in necrosis fit, or are dowelled into small circular holes on the surface of the dead bone. John Hunter investigated the subject of exfoliation as a vital process, and recent investigations with the microscope, have confirmed his observations in a remarkable manner. Chemistry has presented its aid, and it is an important one. Through that aid, we have ascertained the fact that pus discharged from the vicinity of exfoliating bone has in it a large proportion of phosphate of lime. Pus of this kind was found to yield 2.43 per cent. of phosphate of lime, and 2.08 per cent. of carbonate of lime and soda. Thus in the pus from parts around diseased bone, the phosphate of lime amounted to nearly two and a half per cent., while in pus elsewhere obtained, only traces of phosphate of lime were discovered.

*Mode of Removal of Dead Bone.*—There are instances in which dead bone disappears without any sensible exfoliation of it. Mr. Hunter's doctrines induced the supposition that this disappearance, under these circumstances, was the result of the action of the absorbent vessels in the granulations adjacent to the dead bone. On the other hand, it is denied that dead bone is ever absorbed into the system. Miescher has made some observations that seem to disprove the fact of absorption. He has shown that there is a process of exfoliation, in which the dead bone is detached in particles so minute as not to be detected by the unaided sight, and which, therefore, has been termed insensible exfoliation. This view accords with the well ascertained fact of there being a larger quantity of the earthy salts of bone in pus obtained from the neighborhood of exfoliating bone, than in pus made under other circumstances.

Mr. Stanley thinks that when the dead bone is extruded from the cavity in which it was lodged, the process is effected by the pressure of the surrounding granulations against the bone. This extension does not always occur, either in consequence of the size of the dead bone, or from its being enclosed within a tube of the newly formed bone. The next question is whether the exfoliating bone remaining within this cavity, can be acted upon and removed by the adjacent living bone. Sir William Blizzard thought he obtained proof of a removing power in the living bone, but his experiments have been repeated often without the results he claimed, and Mr. Stanley's facts, and Mr. Gulliver's memoir on the subject conclusively show that no power is exerted by the living bone for the removal of the dead one, and wholly disprove the absorption of it. Enfoliated dead bone may acquire adhesion to the living bone: Miesher observed the granulations of the adjacent living bone penetrating the medullary canals of the dead bone.

Exfoliation is variable in respect to the rapidity of its progress, and neither age nor health gives any data on which to found an idea of the time that may be occupied in the process. It commences sometimes and stops without any apparent cause, and sometimes the separation of the dead bone never commences.

Having thus surveyed, through Mr. Stanley's excellent work, the various phenomena that accompany the death of bone, we now turn to the more gratifying contemplation of the efforts of nature at reparation. There is nothing more grateful to the feelings in examining the diseases of the animal economy than a contemplation of these reparative processes, and while following our enlightened guide in these processes, we cannot but regret that one, who has done so much for this whole subject should have failed in any point, and especially in one where the cause of the failure is not altogether obvious. When we reach that point we shall call the attention of the reader to it. We proceed now to consider the reparative processes.

Mr. Stanley says: "If only part of a bone has perished, its remaining portion and the surrounding soft parts become inflamed, and the changes which ensue in these parts constitute the first stage in the process of reproduction. Through its subsequent stages this process is diversely modified, according to the situation and extent of the dead bone, and by other circumstances hereafter to be stated."

"Necrosis in the outer lamella of a bone, when accompanied by destruction of its coverings, including the periosteum, gives rise to the following changes—thickening and consolidation of the inner lamella of the bone, inflammation of the surrounding periosteum, occasioning osseous deposit beneath the membrane, and in its tissue ; and in this way the dead bone becomes circumscribed by a thick projecting border of new osseous substance. In the instances which I have examined, the depression in the surface of a bone, consequent on the exfoliation of its outer lamella, was found filled with fibrous tissue. But in the experiments of Miescher, it appeared that reproduction of the exfoliated outer lamella was effected by ossification of the granulations filling the space between the dead and living bone."

Hunter's experiments on these points were unsatisfactory, because he took pains to do as little injury as possible to the soft parts.

Can the osseous matter of the outer lamella be reproduced without the periosteum ? Experiments upon animals show that it can, but Mr. Stanley says, and we believe correctly, "it is doubtful whether in the human subject, the reproductive power of the tissue of bone is adequate to the formation of so great an extent of new bone," (as is required for a shaft of bone). "In the human subject the reproduction of the outer lamella of a bone can probably be but imperfectly, if at all, effected without the aid of the periosteum."

In some cases the reproduction of bone is effected chiefly by the medullary tissue. If the periosteum and medullary tissue are both preserved, in necrosis of the walls of a bone, the reproduction will chiefly ensue from the periosteum.. "When necrosis attacks the inner lamella of a bone, without extending to its medullary and cancellous tissue, the reparative processes which, under favorable circumstances, may ensue, consist in the consolidation, by osseous deposit, of the medullary tissue, in the thickening and induration of the outer lamella, and in the deposit of osseous substance upon the surface of the bone, beneath the periosteum."

In necrosis of the entire thickness and circumference of the shaft of a bone, through a part or whole of its length, we have to consider four distinct sources of reproduction which may severally be concerned in the formation of the new bone: "these are—1, the periosteum; 2, portions of the original bone detached from its surface and remaining connected with the periosteum; 3, the articular ends of the original bone; 4, the soft tissues around the periosteum, or around the bone if the periosteum has been destroyed. The power of the periosteum for ossification seems to be based on irrefragable evidence. On this point Mr. Stanley says:

"The Italian pathologist, Troja, was, I believe, the first to institute this experiment. I have many times repeated it in dogs, and have thus had the opportunity of observing the phenomena of the reproductive process, which are here enumerated in the order of their occurrence:—inflammation of the periosteum, deposit of purulent fluid between the periosteum and the bone, deposit of gelatinous substance upon the inner surface of the periosteum, hardening and ossification of this gelatinous substance, separation of the dead shaft of the bone from its articular ends, and, lastly, the production of osseous substance from the articular ends, which becoming united with the new bone formed upon the internal surface of the periosteum, the osseous cylinder is thereby completed, and its continuity with the articular ends of the original bone established. Accordingly, in the first stage of the reproductive process, the periosteum is found soft and pulpy, from inflammation of its tissue, and consolidated with the surrounding parts, so as to form with them a vascular bed enclosing the dead bone. Upon the inside of this vascular bed, the new bone is formed. The new bone is lined by a layer of soft and very vascular tissue, which becomes the medullary membrane, with which, under favorable circumstances, the new bone is provided. Dr. Macdonald instituted similar experiments; but, immediately after causing necrosis of the shaft of a bone, he mixed madder with the food of the animal: and on examing the parts three days afterwards, he found the periosteum thickened, with a deposit of gelatinous substance upon its inner surface, in which there were osseous particles tinged by the coloring matter of the madder."

Of the opposite view, the adherence of bone to the periosteum, as the reproducing power, we shall, at present, say nothing.

"Two other sources of reproduction in necrosis of the shaft of a bone are yet to be noticed; namely, the articular ends of the bone, and the surrounding soft parts independently of the periosteum."

New osseous substance is produced from the articular ends of the original bone, but it is only in sufficient quantities to make their union with the newly formed shaft of the bone. That the soft tissues can become the reproductive power of bone, even when the periosteum, with its nuclei of ossification is destroyed, is evident from carefully arranged facts presented by Mr. Stanley and from the experiments of Mr. Russell, of Edinburgh, and of Dr. Macdonald. The circumstances favorable to this mode of reproduction are, that around the dead bone there should be a thick stratum of soft parts, and that the inflammation shall be mild enough to terminate in the effusion of serum or fibrin, rather than suppuration.

When a bone is reproduced by the periosteum, that membrane encloses it, but where the periosteum of the original bone is destroyed, the new bone has for its immediate investment a thick layer of dense cellular tissue, which answers all the purposes of the periosteum. The new bone possesses the entire apparatus of nutrition, arteries, veins, absorbents and nerves. The compensating powers of nature are very wonderful.

The following facts mentioned by Mr. Stanley are of importance, and will be urged again when we come to speak of the bandage. There is a period in these reparative processes, when the articular ends of the original bone are detached from it, but not yet so firmly connected with the newly formed bone as to resist the action of the surrounding muscles. If these articular ends are suffered to approximate, under these circumstances, and permitted to remain so, the new bone will be defective in its proper length. In some instances this deficiency has been to the extent of several inches. But worse still—a spasmodic action of the muscles around the separated shaft of the bone may force its pointed extremity into a contiguous joint, or into a large bloodvessel. Such accidents have happened, but they are impossible when the bandage is properly used.

"The cases of necrosis most favorable for the reproductive process, are those in which the bone suddenly, and at once, completely perishes, and in which there has been no interference with the consequent inflammation in the periosteum and surrounding parts, constituting the first stage of the reproductive process."

"Reproduction after necrosis in any of the flat bones is rare, in some of them it never occurs. It is doubtful whether any of the short, cylindrical, or irregularly shaped bones are ever reproduced."

"Instances of necrosis in early life have occurred, wherein a small portion of the dead bone, not separated from the living bone, has remained unchanged for many years, and the fistulous passages in the soft parts leading to it have become closed. * * * After a lapse of time, it may be a year or longer, a fresh attack of inflammation, followed by abscess, and the re-opening of the fistulous passages, has shown that the original source of irritation still existed. As long as the dead bone irritates the adjacent parts into suppuration, the fistulous passages remain open. But a small portion of dead bone may not irritate, and the fistulous passages may close up.

The following list of the least frequent examples of necrosis is given by Mr. Stanley:

*Lower Jaw*, in a girl aged ten years. The reproduction was perfect. Separation of the ramus with the entire condyle. Perfect reproduction.

*Scapula.* Chopart reports the reproduction of this bone.

*Clavicle.* The entire bone was destroyed by necrosis, and a complete reproduction took place. Two cases are reported of this character.

*Sternum.* Necrosis of a great part of this bone, in which reproduction was in progress.

*Femur*, necrosis and exfoliation of the neck with the shaft of the femur in a child. The recovery was perfect, with but little shortening of the limb.

*Fibula*, necrosis of the shaft; reproduction perfect.

The three last instances are from the museum of St. Bartholomew's Hospital, First series, Nos. 63, 204, 158.

We intended to extend the list of reproduction of bones but our waning space prohibits the fulfilment of the intention.

We have now reached Mr. Stanley's observations on the *treatment of necrosis*, and though we must be succinct in this part of our analysis, we shall endeavor to omit no material feature.

The treatment of superficial necrosis divides itself into two considerations: Can we accelerate the exfoliation of the dead bone? or, shall it be removed by operation?

As a general rule the best applications to the soft parts around dead bone are those of a soothing nature. Stimulating applications, of a moderate character are sometimes useful in producing a sufficient excitement to bring about exfoliation, and mercurial ointment, mixed with soap cerate, is recommended by Mr. Stanley. Exercise of the muscles of the limb, in which the necrosis is situated, is another means of expediting exfoliation. The observations of Mr. Stanley have not induced him to favor the application of acids to the bone, except in some cases of superficial necrosis. He very properly prefers muriatic acid to the sulphuric. The latter makes sulphate of lime, which is injurious.

The removal of the dead bone by operation is inexpedient where a superficial necrosis occurs in bones with a thin covering, and if, after a considerable time, the exfoliation is not advancing. The extent to which the surface of the bone has perished, is indicated by the extent of the separation of the soft parts from it, but this gives no index as to the depth of the necrosis.

"In internal and deeply situated necrosis, when a probe, passed through the fistulous passage in the walls of the bone, discovers the dead bone to be loose, the removal by operation is expedient, and this may be the case even when the dead bone is not completely separated."

If the fistulous passage in the walls of a bone leading to the perished portion of its inner lamella or cancellous texture, is of sufficient extent, the dead bone may be extracted through it. The enlargement of the passage is usually requisite, but it is difficult to do it, on account of the long-continued irritation of an internal sequestrum having thickened and indurated the walls of the bone.

In superficial necrosis, the first thing to be done is the division and separation of the soft parts sufficiently to effect the removal of the dead bone.

In internal necrosis, the hole already existing in the walls of the bone should be the centre of the incision through the soft parts, the requisite portion of the walls being removed by a trephine or other suitable instrument. No more of the wall should be removed than is sufficient to allow the removal of the sequestrum. An aperture of no great size may be sufficient for the removal of a large sequestrum, provided it be conveniently situated for cutting forceps. Heuteloup's stone-crushing forceps, and Civiale's branched stone-perforator have been used with advantage for breaking down the sequestrum.

In necrosis of the shaft of a bone, in its first stage, the treatment must be directed to the control of the inflammation in the surrounding soft parts. The effusion of serum or fibrin is preferable to suppuration, and the inflammation should be confined to the neighborhood of the dead bone, and especial care must be taken that it shall not extend to any of the contiguous joints. Mr. Stanley recommends quietude, the local abstraction of blood from the parts by leeches, and it may be necessary to give saline and antimonial medicines. Poultices and fomentations are also recommended. Although it has been lately uttered to the world that inasmuch as "none but a great poet can criticise a great poet," and this fresh and valuable literary discovery is made the basis of the law, that none but one thoroughly acquainted with surgery must criticise surgical doctrines and practice, we must beg leave to suggest from experience, that quietude, and the proper use of the bandage are worth all the rest of Mr. Stanley's treatment, for the object in view.

If suppuration takes place, and it is generally indicated by shiverings, free incisions must be conveniently made for the escape of matter.

"If there should be constant pain deep in the limb, with occasional attacks of inflammation in the surrounding parts, counter irritation, by an issue, is then indicated —and it may be required as long as the dead bone remains.

The next question is upon the circumstances that make it expedient to resort to an operation for the removal of the dead bone.

When the shaft of the bone is separated from its articular extremities, the formation of the new bone is, in general, so far advanced that the dead bone is enclosed in an osseous case. Absorption was formerly relied upon, for the removal of the dead bone, but since it has been shown that the absorption of exfoliated bone is not a tenable doctrine, the consideration of a resort to an operation is important. An operation should be deferred until there is an assurance that the dead bone is loose. The exfoliated bone may be fixed by the surrounding granulations being imbedded in the excavations of its surface.—Dupuytren advises that with the end of one probe resting against the dead bone, a second probe be introduced into another of the fistulous passages, and its end pressed against the dead bone: if there be mobility, it will make itself evident by the impressions communicated through the probe first introduced. Care must be taken in making incisions through the indurated parts, and as little of the walls of the new bone as possible must be removed.

Necrosis of the cancellous texture of the head of the tibia may demand an operation. This should be resorted to as early as may be practicable, because the knee-joint is in danger. But difficulties may arise. The piece of dead bone may be so near the upper articular surface, that its removal would not be practicable without opening the joint. And it is often impossible to decide upon the precise locality of the dead bone. If the knee-joint should be suffering seriously, amputation may be better than an uncertain operation on the head of the tibia.

Among the long bones, operations for the extraction of dead parts of the shaft, are in general performed with the least difficulty upon the tibia. The operation is usually undertaken before the new bone is completely formed, and it be necessary to separate only some narrow, bridge-like portions of the new bone.

Operations are required in the humerus occasionally, for the removal of dead and exfoliated portions of its shaft, but it is unnecessary for us to describe what must be already understood by our readers.

In necrosis of the lower part of the femur, just above the condyles, we meet with great difficulties. The thickening and consolidation of the soft parts around the bone, the changes in the bone itself, the inflammation of the knee-joint, the danger of wounding the femoral vessels in attempting to use the fistulous passages as guides for incisions to the dead bone, constitute a state of case where amputation may be a less evil, than an attempt to remove the dead bone.

We referred to one point in which Mr. Stanley had failed in his observations on necrosis. He very strangely says that in necrosis of the first and second phalanx of a finger, or of the ungual phalanx, the soft parts should be opened, and the dead bone removed, but that *the bone will not be reproduced.* We cannot conceive a reason for such a statement. All the conditions of reproduction are present—we have periosteum, an articulatory end of bone, and a sufficiency of soft parts, and why is it, that while from the presence of any one of these sources of reproduction elsewhere, we might reasonably look for reparative action, we must not expect it in a case where all three are present? In cases of this kind emollient poultices are generally recommended, after the removal of the dead bone, and a suppurative action is established, which defeats the reparative process. The application of the bandage to the finger or toe, so as to limit the inflammation to a regenerative power, will reproduce bone in these cases, as certainly as it is reproduced in any other part of the system. We speak from a tolerably ample experience, when we say we have never failed to reproduce bone in the phalanges of the fingers. In applying the bandage we must be careful that in the attempt to reproduce bone, we do not make the finger too long nor too short. A little care will secure all the desired benefits.

We have thus moved with Mr. Stanley through the whole of the subject of necrosis, and have found the occupation a pleasant one. Our companion is one of those practical and useful investigators who advance the interests of a profession which is identical with the interests of humanity. He has illustrated the various principles, observations and facts we have quoted from him, by a multitude of interesting cases, which demand the study of the physician.

We have many more valuable things to present our readers from Mr. Stanley's work, but we must defer the pleasure to another time. But we beg leave to call the attention of Messrs. Lea & Blanchard to the necessity they should feel for publishing Mr. Stanley's plates, twenty-four in number, as an accompaniment to this work.—The medical public in this country will feel indebted to them for a publication of such value. Indeed, without it, Stanley on Diseases of the Bones will be what Malte Brun's geography would be without an atlas.

